# Non-HDL-c/TC: A Novel Lipid-Related Marker in the Assessment of Severity of Coronary Artery Lesions and Cardiovascular Outcomes

**DOI:** 10.1155/2019/5931975

**Published:** 2019-04-23

**Authors:** Anan Huang, Xin Qi, Liping Wei, Mingyin Zhang, Shiqi Zhou

**Affiliations:** ^1^Nankai University School of Medicine, 94 Weijin Road, Tianjin, China; ^2^Division of Cardiology, Tianjin Union Medical Center, Nankai University Affiliated Hospital, 190 Jieyuan Road, Hongqiao District, Tianjin, China

## Abstract

**Background:**

Non-high-density lipoprotein cholesterol (non-HDL-c) predicts the severity of coronary artery lesions in patients not treated with statin. The association between non-HDL-c and severity of coronary artery lesions in patients treated with lipid-lowering therapy has been unknown.

**Hypothesis:**

We hypothesize a novel marker of non-HDL-c/TC predicts the severity of coronary artery lesions and clinical outcomes in 12 months in the patients treated with statin.

**Method:**

473 subjects who met inclusion criteria were eligible for inclusion. Coronary artery angiography (CAG) was performed, and the Gensini score (GS) was calculated in all the subjects divided into three subgroups of low risk, medium risk, and high risk by the tertiles of GS. The non-HDL-c value was calculated as TC minus HDL-c, while non-HDL-c/TC was the ratio of non-HDL-c and TC.

**Results:**

The concentration of non-LDL-c differed between non-obstructive-CAD group and obstructive-CAD group (*P* < 0.05), and non-HDL-c/TC was elevated in the obstructive-CAD group (*P* < 0.05). Increased GS was associated with increasing non-HDL-c/TC (*P* < 0.05). Non-HDL-c/TC (OR: 108.50, 95% CI: 1.57–7520.28; *P*=0.030) remained as an independent predicting factor of high risk under GS stratification. In unadjusted Cox model, high non-HDL-c/TC (RR: 1.976, 95% CI: 1.155–3.382; *P*=0.013) predicted the occurrence of adverse events. After multivariate adjustment, high non-HDL-c/TC (RR: 1.921, 95% CI: 1.105–3.339; *P*=0.021) was an independent predictor of poor outcomes.

**Conclusion:**

High level of non-HDL-c/TC presented an excellent prognostic value compared with other lipid-related markers in CAD patients treated with statin.

## 1. Introduction

Cardiovascular disease (CVD), as one of the most common causes of death worldwide, caused 17.3 million deaths worldwide which is more than twice that caused by cancer [1, 2]. In Europe, over 4 million people die of cardiovascular disease (CVD) each year [3]. A total of over 16.5 million Americans aged over 20 years have coronary artery disease (CAD) between 2011 and 2014 with prevalence of 6.3% in U.S. [4].

The link between blood lipids and CAD risks was initially discovered nearly 80 years ago, and many studies centered on the diagnosis or prognosis of CAD suggested dyslipidemia might be associated with severity of atherosclerosis [5, 6]. These results improved our understanding of CAD by suggesting that blood lipid levels increase cardiovascular risk. Recently, multiple risk assessment systems including Framingham model, Systemic Coronary Risk Estimation (SCORE), and Prospective Cardiovascular Münster Study (PROCAM) which included multiple lipid-related markers of total cholesterol (TC) and high-density lipoprotein cholesterol (HDL-c) were recommended to assess total CAD or CV risks in several current national guidelines [7–9].

Multiple guidelines from China, Europe, USA, or Canada recommended that low-density lipoprotein cholesterol (LDL-c) be used as the primary risk estimation for CVD and low HDL-c be an independent risk marker [10–13]. In addition, several novel cholesterol-associated markers such as non-HDL-c and non-HDL-c/HDL-c are also considered as alternative analysis for risk estimation [14]. While statins reduce LDL-c and raise HDL-c, few studies have evaluated cardiovascular risk in persons already treated with statins [11]. Therefore, identifying the best cholesterol-related marker to judge the severity of atherosclerosis seems essential in patients treated with statins.

Non-HDL-c, calculated as the difference between TC and HDL-c, predicts CVD risk equivalent to or more robustly than LDL-c for capturing a more complicated pattern of dyslipidemia in those patients combined with high triglyceride [15, 16].

Previously, literatures have revealed that elevated lipoprotein levels like TG, LDL-c, intermediate HDL-c, and small HDL-c indicated severe coronary artery lesions [11, 17]. However, non-HDL-c is affected by baseline TC level and lipid-lowering drug. Non-HDL-c may not accurately predict the severity of coronary artery lesions in patients already treated with lipid-lowering therapy before. To the best of our knowledge, few studies have studied the association between non-HDL-c and severity of coronary artery lesions in patients treated with lipid-lowering drugs.

In this study, we evaluated the prognostic ability of a novel marker, the ratio of non-HDL-c to TC. We hypothesize that non-HDL-c/TC is a better cardiovascular risk marker in patients treated with statins. Our study aims to compare non-HDL-c/TC with non-HDL-c in predicting the severity of coronary artery lesions and outcomes in 12 months.

## 2. Materials and Methods

### 2.1. Study Design and Population

A clinical retrospective study was designed for evaluating non-HDL-c or non-HDL-c/TC to severity of coronary artery lesions and prognosis of CAD. A total of 629 consecutive individuals with chest pain were evaluated for inclusion in our study between September 2014 and October 2016. 493 subjects were eligible for inclusion. Persons were included in the study if (1) there were clinical findings suggestive of possible CAD including stable angina pectoris, unstable angina pectoris, non-ST segment elevated myocardial infarction (NSTEMI), and ST segment elevated myocardial infarction (STEMI); (2) the patient had coronary angiography performed; (3) medical history including statins at least 3 months before entering this study; and (4) test of lipid metabolism including TC, HDL-c, as well as VLDL-c, or LDL-c. Exclusion criteria included (1) prior PCI therapy, (2) unavailable clinical data especially TC or HDL-c, and (3) comorbidity of thyroid dysfunction, severe liver dysfunction, and/or renal insufficiency or malignant tumor. The Ethical Committee Board of Tianjin Union Medical Center approved this study protocol.

### 2.2. Clinical Data Collection

The demographic characteristics and medical history were recorded at the time of hospitalization. Fasting blood samples were obtained in precooled EDTA and centrifuged at 3600 rpm for over 10 min. Laboratory indices including creatinine kinase (CK), creatinine kinase-MB (CK-MB), high-sensitivity-C reactive protein (hs-CRP), creatinine (Cr), hemoglobin A1c (HbA1c), TC, total triglyceride (TG), HDL-c, LDL-c, and very-low-density lipoprotein cholesterol (VLDL-c) were tested by the biochemistry analyzer (Abbott Architect C-16000 system, Chicago, U.S). Briefly, the TC level was detected by the CHOD-PAP method (cholesterol reagent; Shanghai Fosun Long March Medical Science Co., Ltd) with a coefficient variation (CV) of less than 4%. The GPO-PAP method was performed to test TG (triglycerides reagent; Shanghai Fosun Long March Medical Science Co., Ltd) combined with CV of <5%. In addition, the HDL-c (HDL-cholesterol reagent kit; Shanghai Fosun Long March Medical Science Co., Ltd) and LDL-c (LDL-cholesterol reagent kit; Shanghai Fosun Long March Medical Science Co., Ltd) levels were determined by the clearance method (HDL-cholesterol reagent kit) with a CV of <3% or 4%, respectively. The relative deviation of all kits was not more than 10%. The non-HDL-c value was calculated as TC minus HDL-c, and meanwhile non-HDL-c/TC was the ratio of non-HDL-c and TC.

### 2.3. Evaluation of Severity of Coronary Artery Lesions

Coronary artery angiography (CAG) was performed in all patients using the Judkins technique by 2 experienced cardiac interventional physicians [18]. Based on the coronary artery angiographic results, Gensini score (GS) was calculated in all the participants for quantifying the degree of coronary artery lesions. The specific computing method of GS score has been depicted in the literature previously [19]. Briefly, both the severity of coronary artery stenosis and its geographic location are incorporated in the GS model. All the patients were classified into low-risk, medium-risk, and high-risk subgroups by the tertiles of GS. Coronary stenosis over 50% in one of three main coronary arteries was considered as CAD.

### 2.4. Follow-Up and Outcomes

Clinical follow-up data were obtained by clinic visits every 1 month, telephone interviews every 2 weeks, and analysis of readmission. The primary outcomes included all-cause mortality or cardiovascular mortality. The secondary outcomes included the reoccurrence of chest pain or rehospitalization for PCI or CABG. Follow-up continued until reaching the combined outcomes or censoring on October 31, 2017. Patients showed primary outcome or secondary outcome and were recorded as adverse events.

## 3. Results

**Figure 1:**
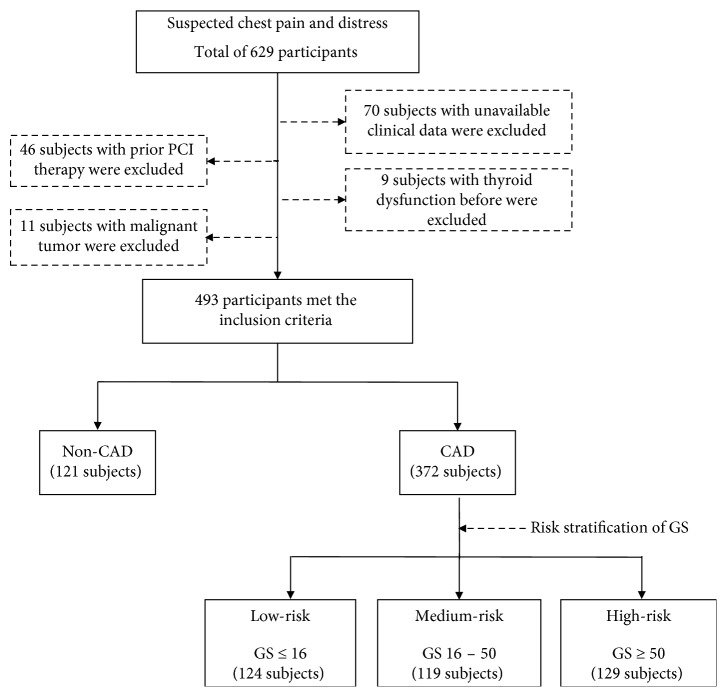
Study flow chart: participant selection in the study. PCI: percutaneous coronary intervention; CAD: coronary artery disease; GS: Gensini score.

From September 2014 through October 2016, a total of 629 patients with suspected chest pain or distress were screened (Figure 1). 493 consecutive individuals were finally included in this study with the exclusion of 136 subjects. Of these, 46 persons had prior PCI, 20 persons had a diagnosis of a malignant tumor or thyroid dysfunction, and 70 persons had incomplete clinical data or no treatment with statins. 493 patients met the inclusion criteria and were divided into non-obstructive-CAD group (121 subjects) or obstructive-CAD group (372 subjects). Patients with <50% of any major epicardial coronary artery were classified into non-obstructive-CAD group while patients ≥50% stenosis were classified as obstructive-CAD group. In the next analysis, patients in obstructive-CAD group were assigned into three subgroups (Low-risk, medium-risk, and high-risk) on the basis of tertiles of GS.

Patients in the non-obstructive-CAD group and different risk subgroups under GS stratification were balanced with regard to the majority of baseline demographic and clinical characteristics well (Table 1). There was no statistically significant different in either group with respect to medical history, and TC, LDL-c, or LVEF levels (*P* > 0.05). The mean age of CAD patients was higher than that of non-obstructive-CAD patients (*P* < 0.05), and the proportion of males increased in the obstructive-CAD group compared to the non-obstructive-CAD group (*P* < 0.05). The laboratory indices including CK, CK-MB, hs-CRP, Cr, HbA1c, TG, and VLDL-c (*P* < 0.05) were increased significantly in patients with CAD. LDL-c level showed no difference between non-obstructive-CAD group and obstructive-CAD group (*P* > 0.05). However, HDL-c levels were lower in patients with CAD (*P* < 0.05). The left ventricular ejection fraction (LVEF) was comparable in both groups (*P* > 0.05). The concentration of non-HDL-c was significantly increased in obstructive-CAD group (*P* < 0.05), and non-HDL-c/TC elevated in obstructive-CAD group compared to non-obstructive-CAD group (*P* < 0.05). The comparison among distinct risk subgroups under the GS stratification was also analyzed. Increased GS was associated with increasing age, male sex, Cr, HbA1c, HDL-c, and non-HDL-c/TC (*P* < 0.05).

Ordered logistic regression analysis was performed for evaluating risk factors for severity of coronary artery lesions. Univariate and multivariate-adjusted RRs are presented in Table 2. On univariate analysis, male, age, HDL-c, HbA1c, and non-HDL-c/TC were possible confounding factors for high GS. After multivariate ordered logistic regression analysis, non-HDL-c/TC (OR: 108.50, 95% CI: 1.57–7520.28; *P*=0.030) remained as independent predicting factor of high risk under GS stratification, as well as male (OR: 2.95, 95% CI: 1.86–4.69; *P* < 0.001), age (OR: 1.05, 95% CI: 1.02–1.08; *P*=0.001), and HbA1c (OR: 1.43, 95% CI: 1.20–1.71; *P* < 0.001), while HDL-c was no longer statistically significant (*P* > 0.05).

The incidence of adverse events was recorded during the 12-month follow-up in our obstructive-CAD group. The baseline characteristics of nonadverse events and adverse events subgroups are shown in Table 3. The percentage of smoking elevated significantly in obstructive-CAD patients with adverse events subgroup with 63.6% (VS nonadverse events subgroup: 45.1%, *P*=0.011); however, the ratio of hypertension decreased with 43.6% (VS nonadverse events subgroup: 43.6% *P*=0.024). No difference was shown in other medical history including DM or dyslipidemia (*P*=0.050, 0.919) as well as male sex (*P*=0.093) and age (*P*=0.827). The laboratory indices are also shown in Table 3; there were no differences in both subgroups except HDL-c (*P*=0.011). In addition, Gensini score and non-HDL-c/TC elevated in adverse event subgroup compared with that of nonadverse events subgroup (*P* < 0.001, 0.031), while no difference was seen in non-HDL-c level and LVEF (*P*=0.785, 0.054).

**Figure 2:**
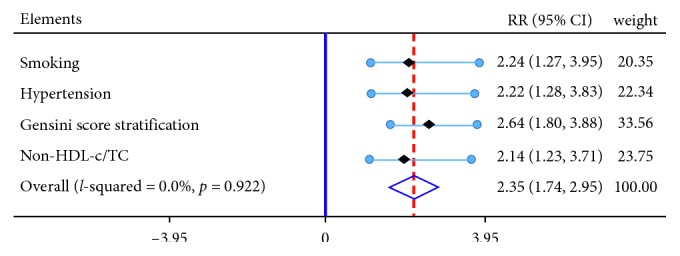
Forest plots of adjusted RR for the adverse events. RR: relative risk; CI: confidence interval.

**Figure 3:**
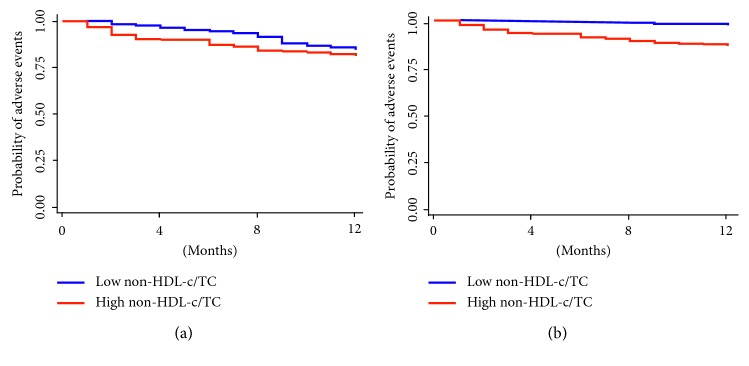
(a) Unadjusted and (b) Adjusted Kaplan–Meier survival curves of non-HDL-c/TC. This model is adjusted for smoking, hypertension, and Gensini score.

The present study suggested non-HDL-c/TC might be an independent factor for predicting the occurrence of adverse events. All the patients with obstructive CAD were divided into high non-HDL-c/TC level or low non-HDL-c/TC based on the median value (0.751) of non-HDL-c/TC. Survival analysis using the Cox regression model was performed to evaluate the independent risk factor for adverse events (Table 4 and Figure 2). In unadjusted Cox model, high non-HDL-c/TC (RR: 1.976, 95% CI: 1.155–3.382; *P*=0.013), smoking (RR: 1.779, 95% CI: 1.024–3.092; *P*=0.041), hypertension (RR: 1.737, 95% CI: 1.020–2.960; *P*=0.042), and Gensini score (RR: 1.779, 95% CI: 1.024–3.092; *P*=0.041) predicted the occurrence of adverse events. After adjusting for these factors, high non-HDL-c/TC (RR: 1.921, 95% CI: 1.105–3.339; *P*=0.021), smoking (RR: 2.276, 95% CI: 1.289–4.022; *P*=0.005), hypertension (RR: 1.873, 95% CI: 1.088–3.227; *P*=0.024), and Gensini score (RR: 1.012, 95% CI: 1.007–1.016; *P* < 0.001) were independent risk factors for predicting poor outcomes. The unadjusted Kaplan–Meier curves presented slight difference (*P*=0.041) of prognosis between high HDL-c/TC and low HDL-c/TC, while this difference was further amplified after adjusting for hypertension, smoking, and GS (*P*=0.017) (Figure 3).

## 4. Discussion

This study shows that, in patients with CAD who are treated with statins, high level of non-HDL-c/TC, while not non-HDL-c, is associated with high GS. Non-HDL-c/TC is an independent risk factor in estimation of severity of coronary atherosclerosis. Accordingly, increased non-HDL-c/TC, which predicts more severe coronary artery lesions, is associated with a poor outcome in 1-year follow-up. After adjusting for several confounders, high level of non-HDL-c/TC indicated poorer prognosis. Overall, these findings support the hypothesis that, in patients treated with statins, non-HDL-c/TC may be a superior predictor of events at one year.

This study focused on the predicting value of lipid-related markers in the assessment of coronary artery lesions and clinical outcomes. In several clinical conditions, series of lipid markers such as TC, LDL-c, HDL-c, and non-HDL-c have been utilized in predicting the risk of CVD [11]. In addition, non-HDL-c and HDL-c can predict the severity of coronary artery lesions. Non-HDL-c indicates a total of cholesterol within all the apolipoprotein B (Apo B) particle including LDL-c and remnant cholesterol [20]. From a prospective study of CGPS, the remnant cholesterol, composed of VLDL-c and intermediate-density lipoproteins (IDL-c), is also a causal risk factor for ischemic heart disease (IHD) [21]. Thus, LDL-c level alone can definitely underestimate the real cardiovascular risk, or severity of atherosclerosis, and further overestimate the prognosis of CAD. Non-HDL-c representing harmful cholesterol could accurately assess the cardiovascular risk and severity of coronary artery lesions in CAD.

However, these lipid-related markers are altered by numerous classes of lipid-lowering drugs which may be one of the confounding factors in predicting severity of coronary artery lesions and short-term clinical outcomes. Statins, as a first line in the treatment of CAD, can significantly reduce serum TC and LDL-c levels or even inhibit VLDL synthesis as well as increase HDL-c levels slightly. In patients with suspected CAD, intensified statins therapy has been recommended by numerous national guidelines [22–24].

Non-HDL-c is calculated as the difference between TC and HDL-c, which can be increased after using statins through reducing LDL-c level. Therefore, statins therapy may underestimate the severity of coronary artery lesions and finally overestimate the clinical prognosis in the real world. A retrospective study enrolled a total of 1757 consecutive patients, and all the participants were divided into four groups based on the GS stratification [17]. Patients with high GS presented elevated non-HDL-c, and non-HDL-c may be a better predictor compared with LDL-c. All the participants in that retrospective study recruited only those treated without any lipid-lowering drugs, thus no confounding from statins insisted on in this study. In the real world, more patients have accepted treatment of statins before CAD. Despite this, the association between lipid-related markers and severity of coronary artery lesions has been unknown in patients treated with statin.

Statin could affect the non-HDL-c and TC value through lowering LDL-c, which may underestimate the cardiovascular risks. Non-HDL-c/TC, as the ratio of non-HDL-c and TC, may weaken this confounding. Non-HDL-c/TC, compared to non-HDL-c, could better reflect the basal lipid level and study the association with severity of coronary artery lesions and clinical outcome in the real world. Although non-HDL-c and non-HDL-c/TC were both increased in patients with CAD in our study, only non-HDL-c/TC differed in the high-risk subgroup. There was no significant difference of non-HDL-c seen among distinct GS risk subgroups. No systemic retrospective study has presented the true association between non-HDL-c level and coronary artery lesions in patients treated with statins.

In our study, high level of non-HDL-c/TC, but not non-HDL-c, showed poor outcomes in 1-year follow-up. Our findings are similar to previously published studies [25, 26]. In those studies, statin might be one of the major confounding factors which could affect the non-HDL-c level. Even though statins were used for all the participants, the ratio of non-HDL-c and TC could weaken a bit of this confound. Patients with non-HDL-c level of over 0.751 might predict poor outcomes.

Several limitations are present in this study. It was only a cross-sectional study of patients with ischemic symptoms and acceptance of CAG; however, those with asymptomatic CAD could not be enrolled into our study. The small sample size and single center preclude application of the study's findings to the general population. Only 1-year follow-up is a relatively short time for assessment of prognosis, and a longer follow-up period will be needed for definitive conclusions.

## 5. Conclusion

This current study supported the hypothesis that non-HDL-c/TC is more useful than non-HDL-c in predicting the severity of coronary artery lesions in patients treated by statin. High level of non-HDL-c/TC had excellent prognostic value compared with other lipid-related markers in CAD patients treated with statin.

## Figures and Tables

**Table 1:** Baseline characteristics of the study population.

	Non-CAD (*n*=121)	CAD			*P*_1_ value	*P*_2_ value
		Low risk (*n*=124)	Medium risk (*n*=118)	High risk (*n*=129)		
Age (years)	59.47 ± 8.07	61.27 ± 8.45	63.11 ± 7.62	63.81 ± 8.04	<0.001^*∗∗*^	0.036^*∗*^
Male (*n* (%))	39 (32.2%)	48 (38.7%)	71 (60.2%)	82 (63.6%)	<0.001^*∗∗*^	<0.001^*∗∗*^
Smoking (*n* (%))	38 (31.4%)	54 (43.5%)	67 (56.8%)	57 (44.2%)	0.001^*∗∗*^	0.056
Medical history (*n* (%))						
Hypertension	61 (50.4%)	67 (54.0%)	64 (54.2%)	83 (64.3%)	0.113	0.166
DM	43 (35.5%)	48 (38.7%)	50 (42.4%)	60 (46.5%)	0.324	0.454
Dyslipidemia	57 (47.1%)	70 (56.5%)	64 (54.2%)	78 (60.5%)	0.194	0.603
Laboratory index						
CK (U/L)	77 (55, 104)	77 (52, 120)	84 (58, 117)	85 (60, 151)	0.038^*∗*^	0.142
CK-MB (U/L)	10 (8, 12)	12 (9, 15)	12 (9, 16)	13 (9, 18)	<0.001^*∗∗*^	0.163
hs-CRP (U/L)	1.01 (0.51, 2.10)	1.82 (0.67, 3.20)	1.40 (0.60, 2.98)	2.28 (0.90, 5.15)	0.001^*∗∗*^	0.057
Cr (mg/dL)	0.67 ± 0.14	0.71 ± 0.16	0.74 ± 0.16	0.81 ± 0.21	<0.001^*∗∗*^	0.001^*∗∗*^
HbA1c (%)	6.11 ± 0.74	6.25 ± 0.98	6.48 ± 1.11	6.85 ± 1.46	<0.001^*∗∗*^	0.001^*∗∗*^
TC (mg/dL)	4.36 (3.87, 5.22)	4.69 (4.13, 5.32)	4.70 (4.11, 5.28)	4.70 (4.11, 5.30)	0.141	0.724
TG (mg/dL)	1.39 (0.99, 1.92)	1.66 (1.10, 2.27)	1.54 (1.18, 1.97)	1.72 (1.24, 2.43)	0.006^*∗∗*^	0.099
HDL-c (mg/dL)	1.17 (1.07, 1.38)	1.19 (1.06, 1.40)	1.15 (0.98, 1.30)	1.08 (0.96, 1.27)	0.006^*∗∗*^	0.005^*∗∗*^
LDL-c (mg/dL)	2.85 ± 0.85	3.03 ± 0.84	3.01 ± 0.86	3.05 ± 0.92	0.241	0.950
VLDL-c (mg/dL)	0.65 (0.45, 0.89)	0.74 (0.52, 1.05)	0.70 (0.54, 0.92)	0.79 (0.57, 1.11)	0.008^*∗∗*^	0.079
LVEF (%)	59 (57, 62)	58 (57, 60)	58 (57, 61)	58 (56, 60)	0.109	0.213
Non-HDL-c (mg/dL)	121.78 (104.58, 154.64)	135.70 (108.34, 159.09)	134.92 (113.47, 155.80)	137.63 (119.65, 158.51)	0.049^*∗*^	0.629
Non-HDL-c/TC	0.74 (0.70, 0.77)	0.75 (0.71, 0.78)	0.75 (0.70, 0.78)	0.76 (0.73, 0.79)	0.006^*∗*^	0.016^*∗*^
Adverse events (*n* (%))	0	7 (5.6%)	16 (13.6%)	32 (24.8%)	NS	<0.001^*∗∗*^

Values are mean ± SD (standard deviation), median (percentiles 25th–75th), or *n* (%). *P*_1_ value indicates comparison among distinct Gensini risk and non-CAD groups. *P*_2_ value indicates comparison among distinct Gensini risk subgroups. ^*∗*^*P* < 0.05 and ^*∗∗*^*P* < 0.01. DM: diabetes mellitus; CK: creatinine kinase; CK-MB: creatinine kinase-MB; hs-CRP: high-sensitivity-C reactive protein; Cr: creatinine; HbA1c: hemoglobin A1c; TC: total cholesterol; TG: total triglyceride; HDL-c: high-density lipoprotein cholesterol; LDL-c: low-density lipoprotein cholesterol; VLDL-c: very-low-density lipoprotein cholesterol; LVEF: left ventricular ejection fraction.

**Table 2:** Independent correlates of severity of coronary artery lesion according to Gensini score stratification.

	Odds ratio	95% CI	*P* value
*Univariate regression*			
Male	2.16	1.47–3.17	<0.001^*∗∗*^
Age	1.03	1.01–1.06	0.012^*∗*^
HDL-c	0.45	0.23–0.88	0.019^*∗∗*^
HbA1c	1.40	1.17–1.67	<0.001^*∗∗*^
Non-HDL-c/TC	138.31	4.67–4095.95	0.004^*∗∗*^
*Multivariate regression*			
Male	2.95	1.86–4.69	<0.001^*∗∗*^
Age	1.05	1.02–1.08	0.001^*∗∗*^
HDL-c	1.48	0.62–3.50	0.376
HbA1c	1.43	1.20–1.71	<0.001^*∗∗*^
Non-HDL-c/TC	108.50	1.57–7520.28	0.030^*∗*^

Multivariate model adjusted for male, age, HDL-c, HbA1c, and Non-LDL. CI = confidence interval. ^*∗*^*P* < 0.05 and ^*∗∗*^*P* < 0.01. HDL-c, high-density lipoprotein cholesterol; HbA1c: hemoglobin A1c.

**Table 3:** Baseline characteristics of CAD patients with adverse and nonadverse events.

	Nonadverse events (*n* = 317)	Adverse events (*n* = 55)	*P* value
Age (years)	62.79 ± 8.12	63.06 ± 7.97	0.827
Male (*n* (%))	177 (55.8%)	24 (43.6%)	0.093
Smoking (*n* (%))	143 (45.1%)	35 (63.6%)	0.011^*∗*^
Medical history (*n* (%))			
Hypertension	190 (59.9%)	24 (43.6%)	0.024^*∗*^
DM	128 (40.4%)	30 (54.5%)	0.050
Dyslipidemia	181 (57.1%)	31 (56.4%)	0.919
Laboratory index			
CK (U/L)	81 (57, 126)	72 (51, 155)	0.993
CK-MB (U/L)	12 (9, 16)	11 (8, 17)	0.964
hs-CRP (mg/L)	1.70 (0.67, 3.27)	1.38 (0.66, 5.17)	0.760
Cr (mg/dL)	0.71 (0.62, 0.87)	0.72 (0.62, 0.85)	0.687
HbA1c (%)	6.1 (5.7, 6.8)	6.3 (5.8, 7.3)	0.063
TC (mg/dL)	175.70 (157.90, 202.01)	181.50 (144.74, 200.85)	0.947
TG (mg/dL)	138.94 (102.66, 184.97)	147.80 (105.32, 181.43)	0.896
HDL-c (mg/dL)	45.67 (39.86, 53.79)	41.80 (37.93, 46.83)	0.011^*∗*^
LDL-c (mg/dL)	114.55 ± 31.35	112.62 ± 27.86	0.684
VLDL-c (mg/dL)	27.48 (20.51, 37.93)	27.09 (20.90, 35.22)	0.792
LVEF (%)	58 (57, 61)	58 (56, 60)	0.054
Gensini score	26 (10, 50)	64 (33, 104)	<0.001^*∗∗*^
Non-LDL-c (mg/dL)	132.47 ± 32.40	133.80 ± 33.07	0.785
Non-LDL-c/TC	0.74 (0.70, 0.78)	0.77 (0.72, 0.78)	0.031^*∗*^

Values are mean ± SD (standard deviation), median (percentiles 25th–75th), or *n* (%). *P* value indicates comparison between nonadverse events and adverse events subgroup. DM: diabetes mellitus; CK: creatinine kinase; CK-MB: creatinine kinase-MB; hs-CRP: high-sensitivity C-reactive protein; Cr: creatinine; HbA1c: hemoglobin A1c; TC: total cholesterol; TG: total triglyceride; HDL-c: high-density lipoprotein cholesterol; LDL-c: low-density lipoprotein cholesterol; VLDL-c: very-low-density lipoprotein cholesterol; LVEF: left ventricular ejection fraction.

**Table 4:** Unadjusted and adjusted survival analysis for predicting adverse events.

Smoking	1.779	1.024–3.092	0.041^*∗*^	2.276	1.289–4.022	0.005^*∗∗*^
Hypertension	1.737	1.020–2.960	0.042^*∗*^	1.873	1.088–3.227	0.024^*∗*^
Gensini score	1.011	1.007–1.016	<0.001^*∗∗*^	1.012	1.007–1.016	<0.001^*∗∗*^
Non-HDL-c/TC (>0.751)	1.976	1.155–3.382	0.013^*∗*^	1.921	1.105–3.339	0.021^*∗*^

Multivariate model adjusted for smoking, hypertension, Gensini score, and non-HDL-c/TC. RR: relative risk; CI: confidence interval. ^*∗*^*P* < 0.05 and ^*∗∗*^*P* < 0.01.

## Data Availability

The original article data used to support the findings of this study are included within the article. Data generated during the study are from clinical data or follow-up information. Several specific data can be accessed by contacting the corresponding author.
